# PC4-mediated Ku complex PARylation facilitates NHEJ-dependent DNA damage repair

**DOI:** 10.1016/j.jbc.2023.105032

**Published:** 2023-07-10

**Authors:** Qimei Pan, Peng Luo, Chunmeng Shi

**Affiliations:** State Key Laboratory of Trauma, Burns and Combined Injury, Institute of Rocket Force Medicine, Third Military Medical University (Army Medical University), Chongqing, China

**Keywords:** DNA damage repair, positive cofactor 4, KU, PARP1, PARylation, nonhomologous end joining, hepatocellular carcinoma

## Abstract

Radiotherapy is one of the mainstay treatments for hepatocellular carcinoma (HCC). However, a substantial number of patients with HCC develop radioresistance and eventually suffer from tumor progression or relapse, which is a major impediment to the use of radiotherapy. Therefore, elucidating the mechanisms underlying radioresistance and identifying novel therapeutic targets to improve patient prognosis are important in HCC management. In this study, using *in vitro* and *in vivo* models, laser microirradiation and live cell imaging methods, and coimmunoprecipitation assays, we report that a DNA repair enhancer, human positive cofactor 4 (PC4), promotes nonhomologous end joining-based DNA repair and renders HCC cells resistant to radiation. Mechanistically, PC4 interacts with poly (ADP-ribose) polymerase 1 and directs Ku complex PARylation, resulting in the successful recruitment of the Ku complex to damaged chromatin and increasing the efficiency of nonhomologous end joining repair. Clinically, PC4 is highly expressed in tumor tissues and is correlated with poor prognosis in patients with HCC. Taken together, our data suggest that PC4 is a DNA repair driver that can be targeted to radiosensitize HCC cells.

Hepatocellular carcinoma (HCC) is one of the most common cancers and the leading cause of tumor-related death worldwide (1). Although surgery is the best treatment for HCC, most patients with HCC are deprived of the chance for resection because the diagnosis is usually made after the optimal time for surgery has elapsed and because of the invasive nature of this treatment. As a result, radiotherapy is increasingly being used for HCC management (2, 3); however, most patients eventually develop resistance to radiotherapy and succumb to the disease, limiting the efficiency of this treatment in HCC (4, 5, 6, 7). Therefore, clarifying the molecular mechanisms underlying radioresistance and identifying novel therapeutic targets are necessary to improve the overall outcomes of patients with HCC.

DNA damage resulting from double-strand breaks (DSBs) is the major mechanism underlying irradiation (IR)-induced cell cycle arrest and apoptosis (8). To maintain chromatin stability and ensure their survival during radiotherapy, cancer cells have developed a complicated protective system called the DNA damage response (DDR) to repair damaged DNA, eventually leading to resistance to radiation (9, 10, 11). Nonhomologous end joining (NHEJ) and homologous recombination (HR) are two crucial DDR pathways (12). As the predominant pathway, NHEJ involves a series of enzymes that capture the ends of broken DNA (*e.g.*, the Ku heterodimer), bring them together (*e.g.*, DNA-PKcs), and ultimately repair the damaged DNA (*e.g.*, DNA ligase IV) (8, 9). The Ku heterodimer, consisting of XRCC5 (Ku80) and XRCC6 (Ku70), is an early-response DDR complex that binds to broken DNA ends and recruits other components to accomplish NHEJ repair (13, 14). The Ku complex is a docking site for DDR-related protein loading; however, whether regulatory intermediates control Ku recruitment to DSB sites remains unknown. Poly (ADP-ribose) polymerase 1 (PARP1) is an abundant, highly conserved DDR protein that is critical for catalyzing poly (ADP-ribosyl) ation (PARylation) of itself and most acceptor proteins at DSB sites (15, 16, 17). PARP1 promotes Ku PARylation and recruitment to DSBs (18). However, the mechanism by which PARP1 regulates the loading of Ku onto DSB sites remains unknown.

Human positive cofactor 4 (PC4) and its yeast ortholog SUB1 are highly conserved nuclear proteins that were initially identified as transcriptional cofactors facilitating RNA polymerase II-driven gene transcription (19, 20, 21). PC4 consists of two major domains: an N-terminal domain, which interacts with proteins, and a C-terminal domain, which is required for binding to single-stranded DNA and double-stranded DNA (22, 23). Specifically, PC4 selectively binds to DNA damaged by platinum (24) and prevents oxidative DNA damage-mediated mutagenesis (23). PC4 also enhances NHEJ-mediated DSB repair by regulating ligase-mediated double-stranded DNA ligation (25, 26) and associates with replication protein A to favor HR repair and promote genome stability (27, 28). These findings imply an important role for PC4 in regulating the DDR pathway. Our studies and others have indicated that the expression of PC4 is upregulated in various cancers, including lung cancer, breast cancer, prostate cancer, astrocytoma, and esophageal squamous cell carcinoma, and that PC4 drives cancer development and progression (25, 29, 30, 31, 32). However, the underlying molecular mechanisms and the functional relevance of PC4 in cancer radiotherapy remain unclear. Additionally, the clinical significance and potential role of PC4 in HCC remain largely unknown.

In this study, we characterized an important regulatory role of PC4 in NHEJ-prone DSB repair in HCC. PC4 is recruited to sites of DNA damage and associates with PARP1, allowing Ku complex PARylation and efficient loading onto DSB sites, thereby facilitating NHEJ repair and enhancing the resistance of HCC cells to IR-induced DNA damage. We believe that these results indicate a reliable biotarget that can be leveraged to suppress resistance to radiotherapy and improve treatment outcomes for patients with HCC.

## Results

### PC4 is a radioresistance factor in HCC

**Figure 1:**
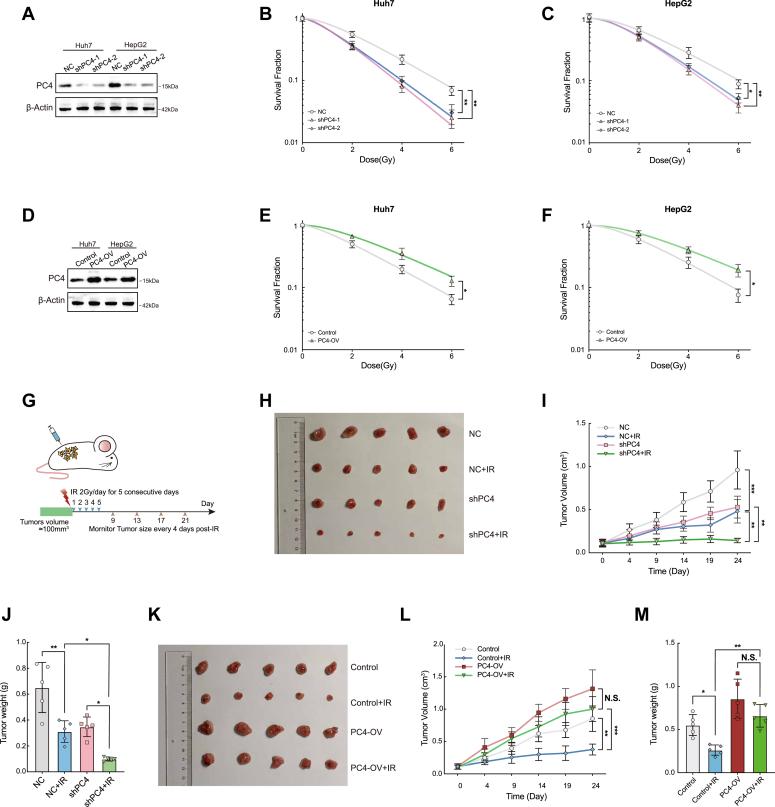
**PC4 is a radioresistance factor in HCC.***A*, Western blot showing PC4 protein levels of Huh7 and HepG2 cells stably expressing control shRNA (shNC), human PC4-targeting shRNA (shPC4-1 and shPC4-2). β-actin, loading control. *B*, the responses of survival factions of Huh7 cells to X-ray irradiation with or without PC4 knockdown. n = 3. *C*, the responses of survival factions of HepG2 cells to X-ray irradiation with or without PC4 knockdown. n = 3. *D*, Western blot showing PC4 protein levels of Huh7 and HepG2 cells stably overexpressing control or PC4 (PC4-OV). β-actin, loading control. *E*, the responses of survival factions of Huh7 cells to X-ray irradiation with or without PC4 overexpression. n = 3. *F*, the responses of survival factions of HepG2 cells to X-ray irradiation with or without PC4 overexpression. n = 3. *G*, the schema of the animal experiments. *H*–*J*, the radiotherapy efficacy of PC4 knockdown in HCC xenografts in nude mice. The dissected xenografts were photographed in indicated groups (*H*). Tumor growth curves were measured every 4 days after IR in indicated groups (*I*). Tumors were weighed at the endpoint in indicated groups (*J*). n = 5 in each group. *K*–*M*, the radiotherapy efficacy of PC4 overexpression in HCC xenografts in nude mice. The dissected xenografts were photographed in indicated groups (*K*). Tumor growth curves were measured every 4 days after IR in indicated group (*L*). Tumors were weighed at the endpoint (M). n = 5 in each group. All data indicate the mean ± SD. ∗*p* <0.05, ∗∗*p* < 0.01, ∗∗∗*p* < 0.001. HCC, hepatocellular carcinoma; PC4, human positive cofactor 4.

Recent studies have described the association of PC4 with genome stability and cancer development (27, 29, 30, 31). Therefore, we speculated that PC4 might enhance the resistance of tumor cells to consecutive insults that cause genotoxic stress. As diverse responses are exhibited after radiotherapy and resistance develops in a large proportion of patients with HCC, we determined whether PC4 is a radiotherapy resistance factor in HCC. To investigate the effect of PC4 on radiosensitivity, we stably knocked down PC4 in Huh7 and HepG2 cells by lentiviral transfection (Fig. 1*A*) and analyzed its effects on cell proliferation after IR. The surviving fraction of cells after IR in the PC4 knockdown group was significantly reduced compared with that in the control group, indicating that PC4 depletion increased the radiosensitivity of HCC cells (Fig. 1, *B* and *C*). In contrast, PC4 overexpression (PC4-OV) induced a statistically significant increase in radiation resistance in both Huh7 and HepG2 cells (Fig. 1, *D*–*F*). To evaluate the role of PC4 in DDR *in vivo*, we generated xenograft tumor models inoculated with Huh7 PC4 WT cells (NC) and PC4 knockdown cells (shPC4). We found that PC4 knockdown reduced tumor growth, suggesting that PC4 influences cell proliferation. The mice were then exposed to X-ray (2 Gy/day for 5 consecutive days) until the tumor volume reached 100 mm^3^ (Fig. 1*G*). Interestingly, radiotherapy markedly decreased tumor growth and weight in the PC4 knockdown group compared with that in the NC group (Fig. 1, *H*–*J*). In contrast, PC4-OV enhanced tumor growth after IR (Fig. 1, *K*–*M*), suggesting that PC4 confers radioresistance to HCC cells. Collectively, these results support the hypothesis that PC4 is associated with radioresistance and may influence the efficiency of DNA damage repair.

### PC4 is immediately recruited to DNA DSBs and promotes DSBs repair

**Figure 2:**
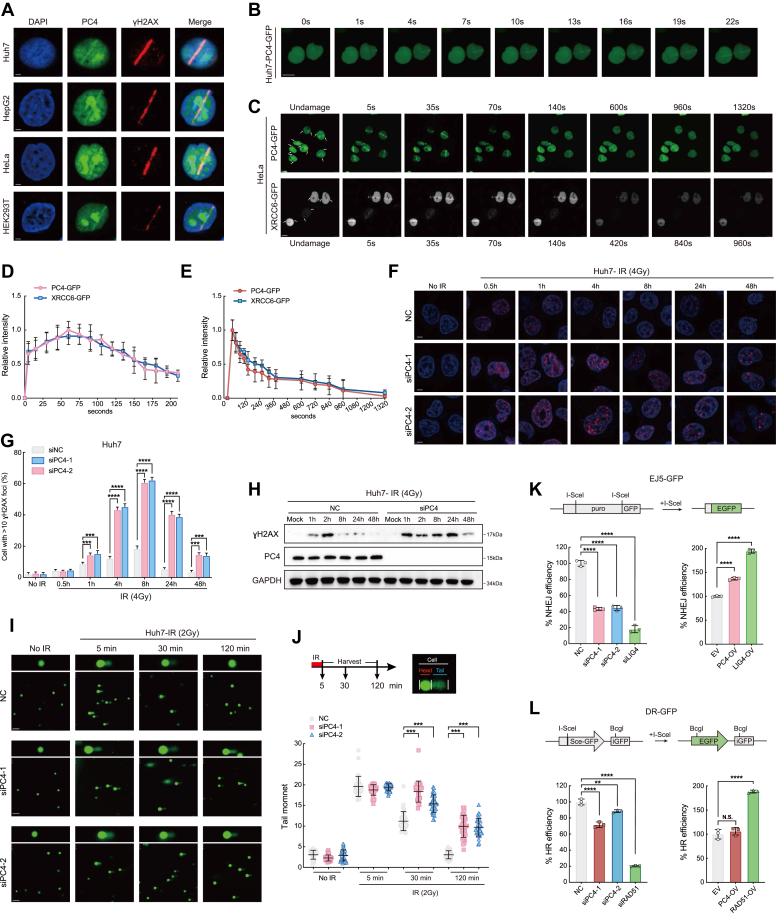
**PC4 is recruited to the DSB sites andpromotes DSBs repair.***A*, endogenous PC4 recruitment to DNA double strand breaks. Huh7, HepG2, HeLa, or HEK293T cells were subjected to laser microirradiation and analyzed by immunofluorescence staining with antibody against PC4 and γH2AX. Scale bars, 2.5um. *B*, representative time-lapse images showing the localization of GFP-PC4 after laser microirradiation in Huh7 cells. Scale bar, 10um. *C*, representative time-lapse images showing the recruitment of GFP-PC4 to multiphoton tracks in HeLa cells. GFP-XRCC6 was used as a DNA damage response marker. Scale bar, 10 μm. *D* and *E*, quantification of GFP-PC4 and GFP-XRCC6 fluorescence intensity at DNA damage sites in *C*. Results shown are typical of four independent experiments and represent 40 different cells. *F*, representative fluorescence images of γ-H2AX immunostaining in Huh7 cells with PC4 knockdown upon irradiation. Scale bars, 5 μm; *red*, γ-H2AX; *blue*, DAPI. *G*, quantification of *F*. n = 3 biologically independent samples. *H*, Western blot analysis of the expression of γ-H2AX in Huh7 cells with PC4 knockdown upon irradiation. *I* and *J*, representative fluorescence images (*J*: scale bars,100 μm) and quantification (*K*: n = 3) of tail moments in Huh7 cells with PC4 knockdown as determined by a neutral comet assay. *K*, characterization the effect of PC4 knockdown or overexpression on NHEJ repair efficiency by EJ5-GFP in Huh7 cells. *Upper*, Schematic representation of NHEJ reporter. *Lower*, the percentage of GFP+ cells was analyzed by fluorescent activated cell sorting, and fold changes were normalized to cells transfected with NC or EV. LIG4 knockdown suppresses the NHEJ repair, while LIG4 overexpression enhances the NHEJ repair, which is represented as a positive control. *L*, characterization the effect of PC4 knockdown or overexpression on HR repair efficiency by DR-GFP. *Upper*, schematic representation of HR reporter. RAD51 knockdown suppresses the HR repair, while RAD51 overexpression enhances the HR repair, which is represented as a positive control. All graphed data were shown as means ± SD. ∗∗*p* < 0.01.∗∗∗*p* < 0.001.∗∗∗∗*p* < 0.0001. DSBs, double-strand breaks; HCC, hepatocellular carcinoma; HR, homologous recombination; NHEJ, nonhomologous end joining; PC4, human positive cofactor 4.

To determine whether PC4 is involved in DNA damage repair, we investigated the recruitment of endogenous PC4 to DNA damage sites. Four different cell lines (Huh7, HepG2, HeLa, and HEK293T) were subjected to UV laser micro-IR and immunofluorescence staining. Endogenous PC4 colocalized with γH2AX at DNA damage sites in all four cell lines (Fig. 2*A*), supporting the hypothesis that PC4 acts as a sensitive sensor for DDR. Next, we examined the kinetics of PC4 recruitment to the sites of DNA damage by live imaging of cells stably expressing PC4-GFP. Consistent with the aforementioned results, PC4 immediately accumulated at the sites of DNA damage within 1 s (Fig. 2*B*); the level peaked at 1 min and slowly decreased over the subsequent 20 min. Moreover, the rapid recruitment followed by the transient retention of PC4 at DNA damage sites was comparable to the dynamics of XRCC6 recruitment after UV laser micro-IR (Fig. 2, *C*–*E*). These findings indicate that PC4 is recruited early in the DDR and can be recruited to UV laser-induced DNA damage sites.

To determine how PC4 regulates DNA damage repair, we explored the effect of PC4 knockdown on the formation of γH2AX foci in Huh7 cells after IR. As expected, in normal cells, 30 min after IR, γH2AX foci were visible, and the number of foci gradually increased, reaching a plateau within 4 h and vanishing within 24 h. In contrast, PC4 knockdown induced the formation of a markedly high number of γH2AX-positive foci 1 h after IR, and the difference remained significant even after 48 h (Fig. 2, *F* and *G*), suggesting that without PC4, the cells failed to efficiently repair DNA damage. Moreover, the sustained γH2AX protein levels in PC4 knockdown Huh7 and HepG2 cells (Figs. 2*H* and S1*A*) confirmed that silencing PC4 impaired the efficacy of DNA DSB repair. Next, we performed a DNA-neutral comet assay to measure the efficiency of DSB repair in PC4 knockdown cells. Notably, the initial degree of DSB induction (5 min after IR) was not affected by PC4 depletion. However, after 2 h of recovery following IR, the DSB repair efficiency in PC4 knockdown cells was much lower than that in control cells, as indicated by the significant increase in the lengths of the comet tails (Fig. 2, *I* and *J*). When cells undergo stress-induced DNA damage, some DDR-related molecules, such as ATM, ATR, CHK2, and CHK1, are phosphorylated, initiating cell cycle arrest and facilitating DNA repair (33). In this study, we observed that PC4 depletion caused a decrease in the expression levels of p-ATM, p-ATR, p-CHK1, and p-CHK2 but had little effect on that of p-DNA-PKcs in Huh7 and HepG2 cells after IR (Fig. S1*B*), suggesting an essential role for PC4 in activating ATM-CHK2 and ATR-CHK1 signaling during DSB repair. To identify the DNA DSB repair pathway mediated by PC4, two DSB reporters, EJ5-GPF and DR-GFP, which represent the activities of NHEJ and HR, respectively, were employed. Depletion of PC4 in both EJ5-GFP- and DR-GFP reporter-expressing cells caused a marked reduction in the number of GFP-positive cells compared to that in control cells (Fig. 2, *K* and *L*). Furthermore, PC4-OV significantly increased the efficiency of NHEJ (Fig. 2*K*) but only slightly increased the efficiency of HR (Fig. 2*L*), indicating that PC4 was required for DSB repair, which was realized mainly through the NHEJ pathway.

### The PC4 C-terminal domain physically associates with XRCC5/XRCC6

**Figure 3:**
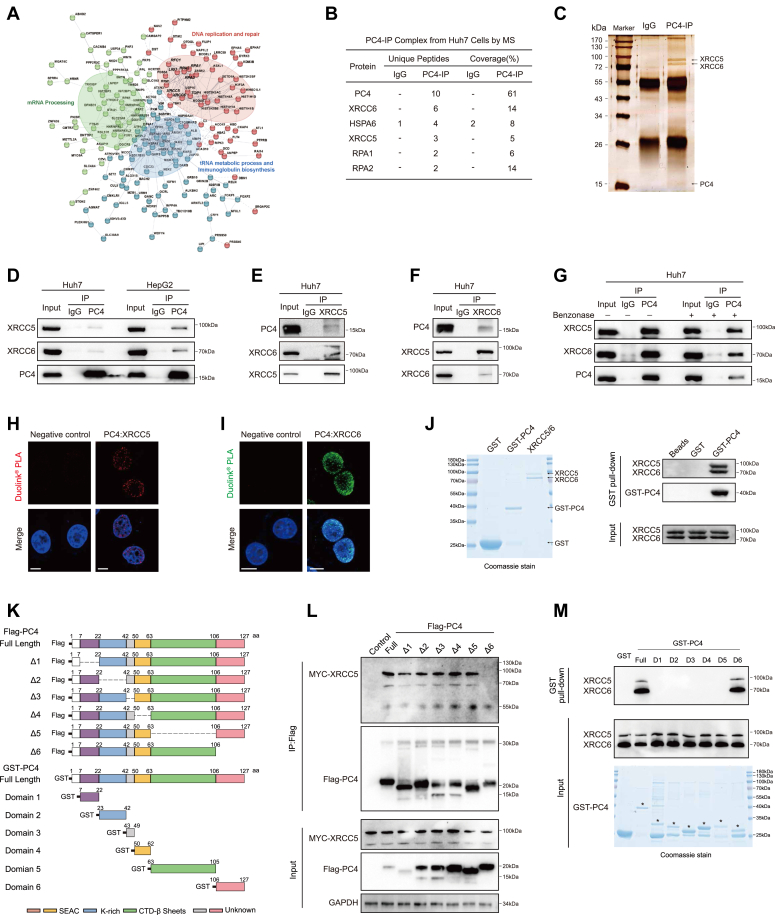
**PC4 physically interacts with Ku complex through C-terminal domain.***A*, visual representation of PC4 interacting proteins using k-means clustering on STRING database (https://string-db.org/). *B*, mass spectrometry analysis of the differential binding proteins of PC4 in Huh7 cells. The parameters of the top five protein candidates are shown. *C*, silver staining of endogenous PC4 complex separated by SDS-PAGE in Huh7 cells. PC4 interacting proteins, including XRCC5 and XRCC6, are indicated. *D*, immunoprecipitation (IP) analysis of the association of PC4 with XRCC5 and XRCC6 in Huh7 and HepG2 cells. *E*, reciprocal co-IP analysis of the association of XRCC5 with PC4 and XRCC6 in Huh7 cells. *F*, reciprocal co-IP analysis of the association of XRCC6 with PC4 and XRCC5 in Huh7 cells. *G*, IP analysis of the association of PC4 with XRCC5 and XRCC6 in Huh7 cells with or without benzonase nuclease. *H*, analysis of PC4 and XRCC5 colocalization with PLA in Huh7 cells by confocal fluorescence microscopy. Negative control represents the sample incubated without PC4 and XRCC5 antibodies, scale bar = 8 μm. *I*, analysis of PC4 and XRCC6 colocalization with PLA in Huh7 cells by confocal fluorescence microscopy. Negative control represents the sample incubated without PC4 and XRCC6 antibodies, scale bar = 10 μm. *J*, GST pull-down assay with GST-tagged recombinant PC4 and XRCC5-XRCC6 complex. The recombinant proteins were examined by Coomassie brilliant blue staining. *K*, schematic representation of Flag-tagged full-length and different truncated mutants of PC4, GST-tagged full-length and different domains of PC4. SEAC: serine/acidic residue-rich region, K-rich: lysine-rich region, CTD-β Sheet: C-terminal β sheet region. *L*, IP analysis of the association of Myc-tagged XRCC5 with FLAG-tagged PC4 deletion mutants in Huh7 cells. *M*, GST pull-down assay with different GST-tagged recombinant PC4 domains and recombinant XRCC5-XRCC6. The recombinant GST-tagged PC4 domains were examined by Coomassie brilliant blue staining. DSBs, double-strand breaks; HCC, hepatocellular carcinoma; HR, homologous recombination; NHEJ, nonhomologous end joining; PC4, human positive cofactor 4; PLA, proximity ligation assay.

To illustrate the regulatory mechanism of PC4 in DNA damage repair, we examined the PC4 interactome. We performed immunoprecipitation (IP) assays with Huh7 cells and subsequently applied mass spectrometry (MS) for endogenous PC4 co-IP (Co-IP). This approach revealed that 1% of the identified proteins showed the highest interaction with PC4, and these partners were broadly clustered under three functional groups: “mRNA processing”, “DNA replication and repair”, and “tRNA metabolic process and immunoglobulin biosynthesis” (Fig. 3*A*). Among the proteins binding to PC4, the XRCC5/XRCC6 complex was one of the most promising candidates related to the DDR pathway (Fig. 3*B* and Table S1). We then subjected the PC4-interacting proteins to SDS-PAGE and silver staining. The results revealed that PC4 may be associated with XRCC5/XRCC6, consistent with the MS results (Fig. 3*C*). XRCC5 interacts with XRCC6 to form an obligate Ku heterodimer, which plays a key role in the NHEJ pathway. Hence, we speculated that PC4 might interact with DNA at DSB sites prone to NHEJ repair through its association with the Ku complex. To verify the relationship between PC4 and XRCC5/XRCC6, endogenous PC4 and XRCC5/XRCC6 were co-immunoprecipitated in Huh7, HepG2, HeLa, and HEK293T cells. The results confirmed the association of PC4 with XRCC5/XRCC6 in relevant cancer and normal cells (Figs. 3, *D*–*F* and S2, *A* and *B*). Moreover, PC4 colocalized with XRCC5 and XRCC6 in the nucleus, as reflected by coexistence of the PC4-XRCC5/XRCC6 proximity ligation assay (PLA) signal (Fig. 3, *H* and *I*). Interestingly, the association of PC4 with the XRCC5/XRCC6 complex was independent of the DNA structure, as indicated by the fact that the addition of benzonase (a nuclease) did not disrupt their interaction (Fig. 3*G*). We also performed an *in vitro* GST pull-down assay using recombinant GST-tagged PC4 and recombinant XRCC5/XRCC6 complex. In line with the aforementioned observations, XRCC5/XRCC6 was pulled down by GST-PC4 (Fig. 3*J*), demonstrating a direct protein–protein association. To gain deeper molecular insights into the PC4-XRCC5 association, we generated a panel of Flag-tagged PC4 deletion mutants and subjected them to Co-IP (Fig. 3*K*). Although the nuclear location of PC4 was not altered by deletion of any domain (Fig. 3*L*), PC4^Δ6^ failed to bind to XRCC5, indicating that the C-terminal domain is critical for the association of PC4 with XRCC5. Furthermore, we purified six truncates of PC4 according to their domains (Fig. 3*K*) and found that only the C-terminal domain of PC4 was associated with the recombinant XRCC5/XRCC6 complex (Fig. 3*M*). These results strongly support the hypothesis that PC4 physically associates with XRCC5/XRCC6 *via* its C-terminal domain and that it is likely to regulate XRCC5/XRCC6 activity in NHEJ repair.

### PC4 is required for XRCC6 PARylation at DSB sites

**Figure 4:**
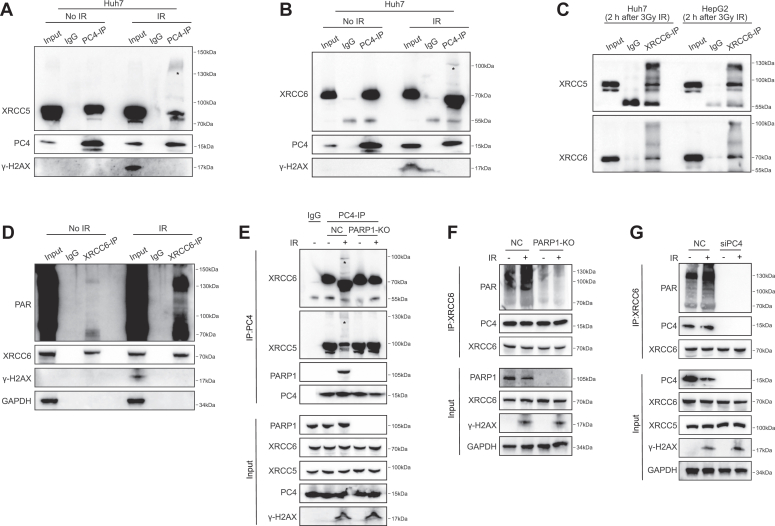
**PC4 is required for Ku complex PARylation at DSB sites.***A*, IP analysis of the association of PC4 with unmodified and modified XRCC5 in Huh7 with or without 3 Gy irradiation. ∗Represents modified XRCC5. *B*, IP analysis of the association of PC4 with unmodified and modified XRCC6 in Huh7 with or without 3 Gy irradiation. ∗Represents modified XRCC6. *C*, IP analysis of XRCC5 and XRCC6 modifications in Huh7 and HepG2 cells after 3 Gy irradiation. *D*, IP analysis of XRCC6 PARylation in Huh7 cells with or without irradiation. *E*, IP analysis of the association of PC4 with modified XRCC5 and XRCC6 in PARP1-knockout Huh7 cell with or without irradiation. *F*, IP analysis of XRCC6 modifications in PARP1-knockout Huh7 cells with or without irradiation. *G*, IP analysis of XRCC6 PARylation in PC4-knockdown Huh7 cells with or without irradiation. DSBs, double-strand breaks; IP, immunoprecipitation; PARP1, poly (ADP-ribose) polymerase 1; PC4, human positive cofactor 4.

To understand how PC4 coordinates the function of XRCC5 and XRCC6 in DSB repair, we first characterized the IR-induced PC4-XRCC5/XRCC6 association. Surprisingly, we found that PC4 bound to the modified XRCC5/XRCC6 upon IR, as evidenced by the higher molecular weight of XRCC5 or XRCC6 immunoprecipitated with PC4 (Figs. 4, *A* and *B* and S3, *A* and *B*). Furthermore, the IR-induced modification of the Ku complex was confirmed in both Huh7 and HepG2 cells (Fig. 4*C*). However, the Ku complex did not bind to the modified PC4 after DNA damage was induced (Fig. S3, *C*–*E*). Because the formation of XRCC5/XRCC6 heterodimers is obligatory, in subsequent investigations, we analyzed only the effects of PC4 on the activity of XRCC6 in NHEJ repair. Given that DSBs cause XRCC6 PARylation, which directs XRCC6 recruitment and retention at damage sites and thus promotes NHEJ repair (18), we reasoned that the modification type induced in XRCC6 by genotoxic insults could be PARylation and that PC4 might mediate the PARylation of XRCC6 after IR-induced DNA damage. Indeed, when Huh7 cells were subjected to IR, a noticeable increase in the PARylation of endogenous XRCC6 was observed (Fig. 4*D*). In contrast, PARP1 depletion significantly inhibited IR-induced XRCC6 modification (PARylation) (Fig. 4, *E* and *F*), indicating that the PARylation of XRCC6 is induced in an IR-dependent manner. Notably, PC4 knockdown resulted in a substantial decrease in IR-induced PARylation of XRCC6 (Fig. 4*G*). Therefore, PC4 was critical for DNA damage-dependent XRCC6 PARylation.

### Direct association of PC4 with PARP1 promotes XRCC5/XRCC6 PARylation

**Figure 5:**
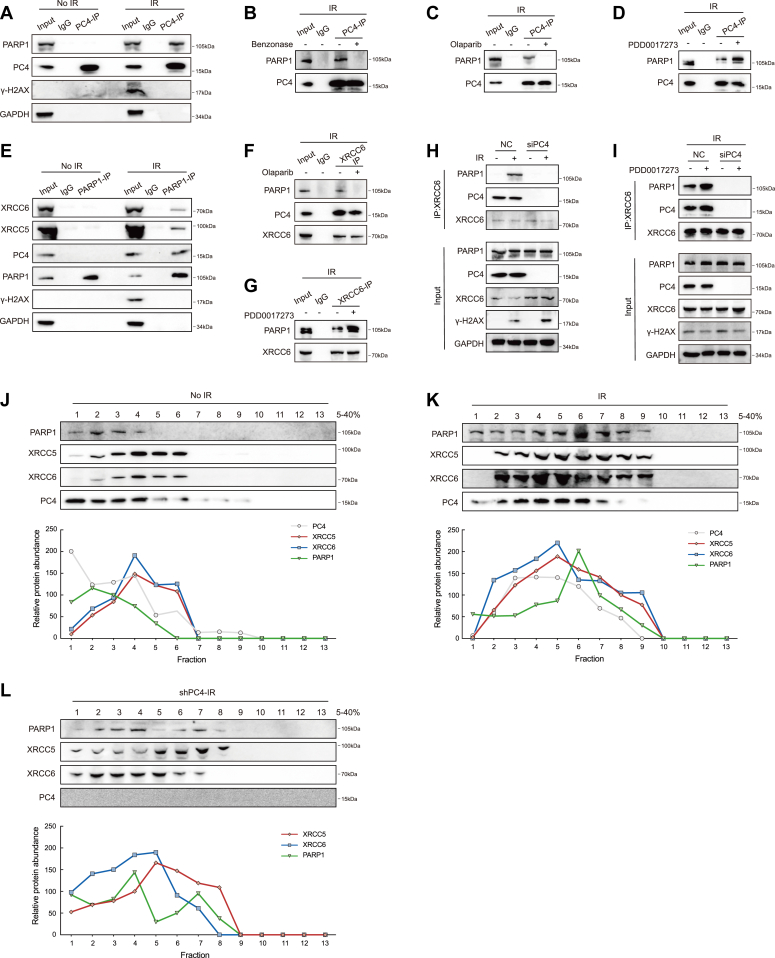
**Direct association of PC4 with PARP1 promotes PARylation of XRCC6 on DSB sites.***A*, IP analysis of the association of endogenous PC4 with PARP1 in Huh7 cells with or without irradiation. *B*, IP analysis of the association of PC4 with PARP1 in Huh7 cells with or without benzonase nuclease after irradiation. *C*, IP analysis of the association of endogenous PC4 with PARP1 in Huh7 cells with or without Olaparib (10 μM) after irradiation. *D*, IP analysis of the association of endogenous PC4 with PARP1 in Huh7 cells with or without PARG inhibitor PDD00017273 (10 μM) after irradiation. *E*, IP analysis of the association of endogenous PARP1 with XRCC5 and XRCC6 in Huh7 cells with or without irradiation. *F*, IP analysis of the association of XRCC6 with PC4 and PARP1 in Huh7 cells with or without Olaparib (10 μM) after irradiation. *G*, IP analysis of the association of XRCC6 with PC4 and PARP1 in Huh7 cells with or without PARG inhibitor PDD00017273 (10 μM) after irradiation. *H*, IP analysis of the association of XRCC6 with PARP1 in PC4-knockdown Huh7 cells with or without irradiation. *I*, IP analysis of the association of XRCC6 with PARP1 in PC4-knockdown Huh7 cells with or without PARG inhibitor PDD00017273 (10 μM) after irradiation. *J*, fractions of Huh7 cells were separated by sucrose gradient centrifugation. The amounts of the indicated proteins were quantified using ImageJ. *K*, fractions of Huh7 cells with 3 Gy irradiation were separated by sucrose gradient centrifugation. The amounts of the indicated proteins were quantified using ImageJ. *L*, fractions of PC4-knockdown Huh7 cells with 3 Gy irradiation were separated by sucrose gradient centrifugation. The amounts of the indicated proteins were quantified using ImageJ. DSBs, double-strand breaks; IP, immunoprecipitation; PARP1, poly (ADP-ribose) polymerase 1; PC4, human positive cofactor 4.

Next, we sought to determine the mechanism by which PC4 mediates IR-induced XRCC6 PARylation. It is well known that in response to DSBs, PARP1 is activated and uses NAD+ as a substrate to poly-ADP (ribosyl)ate a variety of target proteins and contributes to DNA damage repair (15, 16). For example, PARP1 is a PAR writer of XRCC6 that facilitates its loading onto DSB sites (18), which raises the question of whether PC4 recruits PARP1 to rapidly induce the PARylation of XRCC6 on DSBs. To answer this question, we performed co-IP and found that PARP1 was associated with PC4 under IR-induced DNA damage conditions but not under normal conditions (Fig. 5*A*). This association was mediated by DNA (Fig. 5*B*) and relied on PARP activity, as shown by experiments in which the treatment of cells with the PARP inhibitor olaparib before IR prevented PARP1 binding to PC4 (Fig. 5*C*). However, administration of the poly(ADP-ribose) glycohydrolase inhibitor PDD00017273, which reversed the degradation of the poly(ADP)ribose chain, markedly enhanced IR-induced PC4-PARP1 binding (Fig. 5*D*). In addition, DNA damage induced a PARP1-XRCC5/XRCC6 association (Fig. 5*E*), consistent with previous studies, and this association was also dependent on PARP activity (Fig. 5, *F* and *G*). Importantly, PC4 depletion hindered IR-induced PARP1-XRCC6 association (Fig. 5*H*), even though PARP1 was hyperactivated (Fig. 5*I*), illustrating that PC4 is necessary for the PARP1 interaction with XRCC6 and mediates XRCC6-PAR binding, contributing to the repair of DNA damage. To further validate the dynamic association among PC4, Ku complex, and PARP1, we performed sucrose gradient fractionation of Huh7 cell extracts and found that a substantial portion of the Ku complex cosedimented with PC4 in the small-molecular-weight fraction (Fig. 5*J*). Interestingly, when cells were exposed to IR, the Ku complex predominantly comigrated with PC4 and PARP1 in the middle-molecular-weight fraction (Fig. 5*K*). However, PC4 deletion dramatically reduced the presence of the Ku complex in the middle-molecular-weight fraction. In addition, the PARP1 and Ku complexes were present in a relatively different fraction (Fig. 5*L*). Together, these data strongly suggest that PC4 facilitates PARP1-mediated Ku PARylation after IR-induced DNA damage.

### PC4-mediated XRCC6 PARylation promotes XRCC6 recruitment to DSB sites

**Figure 6:**
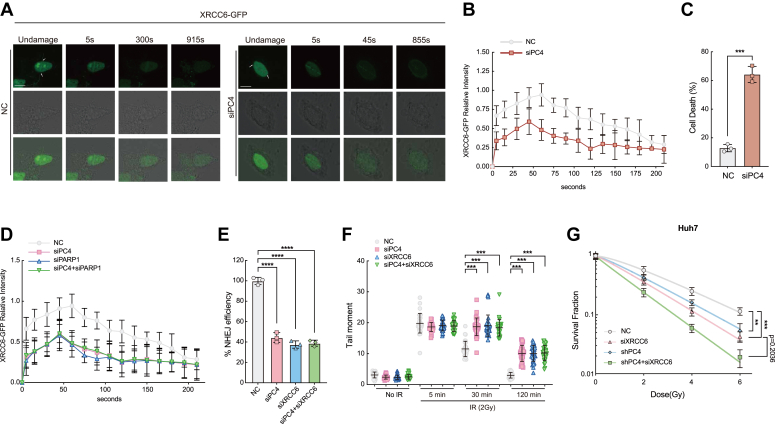
**PC4-mediated XRCC6 PARylation promotes XRCC6 efficient loading on DSB sites.***A*, representative time-lapse images showing the recruitment of XRCC6-GFP to multiphoton tracks in Huh7 cells with or without PC4 knockdown. Scale bar, 10 μm. *B*, quantification of XRCC6-GFP fluorescence intensity at DNA damage sites in *A*. Data were derived from three independent experiments. In each experiment, 50 different cells were investigated. *C*, quantification of cell death in XRCC6-GFP cells upon laser microradiation with or without PC4 knockdown. n = 3. *D*, quantification of XRCC6-GFP fluorescence intensity at DNA damage sites in NC, siPC4, siPARP1, or siPC4+siPARP1 group. Data were derived from three independent experiments. *E*, characterization of the NHEJ repair efficiency by EJ5-GFP and FACS in NC, siPC4, siPARP1, or siPC4+siPARP1 group. n = 3. *F*, quantification of tail moments in NC, siPC4, siPARP1, or siPC4+siPARP1 group as determined by a neutral comet assay. n = 3. *G*, the responses of survival factions of Huh7 cells to X-ray irradiation in NC, shPC4, siXRCC6, or shPC4+siXRCC6 group. n = 3. All graphed data were shown as means ± SD. ∗∗*p* < 0.01, ∗∗∗*p* < 0.001, ∗∗∗∗*p* < 0.0001. DSBs, double-strand breaks; FACS, fluorescent activated cell sorting; NHEJ, nonhomologous end joining; PC4, human positive cofactor 4.

Given that PC4 governs the association of XRCC6 with PARP1 and the subsequent XRCC6 PARylation, we hypothesized that PC4 influences the recruitment of XRCC6 to DSB sites. Indeed, the depletion of PC4 significantly reduced XRCC6 accumulation at sites of DNA damage (Fig. 6, *A* and *B*). Because of the reduction in XRCC6 recruitment after PC4 knockdown, an increased number of cells failed to overcome DNA damage and eventually died (Fig. 6*C*). To corroborate these findings, we examined the interaction between PC4 and PARP1 during XRCC6 loading at DNA damage sites. When either PC4 or PARP1 was depleted, XRCC6 loading was inhibited, which was in agreement with the aforementioned observations. Dual depletion of PC4 and PARP1 did not result in an additive inhibitory effect on XRCC6 accumulation compared to that induced by PC4 or PARP1 knockdown in Huh7 cells (Fig. 6*D*), indicating that PC4 and PARP1 act epistatically to recruit XRCC6 to DNA break sites. Moreover, knockdown of PC4 or XRCC6, alone and in combination, significantly inhibited NHEJ-dependent DSB repair (Fig. 6, *E* and *F*). A similar trend was observed in the survival analysis (Fig. 6*G*), implying that PC4 and XRCC6 function *via* the same epistatic pathway to promote DSB repair by NHEJ.

Taken together, these findings led us to propose that PC4 acts as an important anchor for Ku in NHEJ repair because it (1) senses and binds to DNA damage sites (2), associates with PARP1 in DSBs (3), guides PARP1-mediated Ku PARylation, and (4) provides a structural docking platform for efficient loading of Ku onto DSBs. The PC4–PARP1 complex is cleared from damaged chromatin, and then, the Ku heterodimer recruits downstream executors to realize NHEJ repair. Therefore, the DNA damage-induced PC4-mediated PARylation of the Ku complex is critical for DNA DSB repair.

### PC4 levels might predict outcomes for patients with HCC

**Figure 7:**
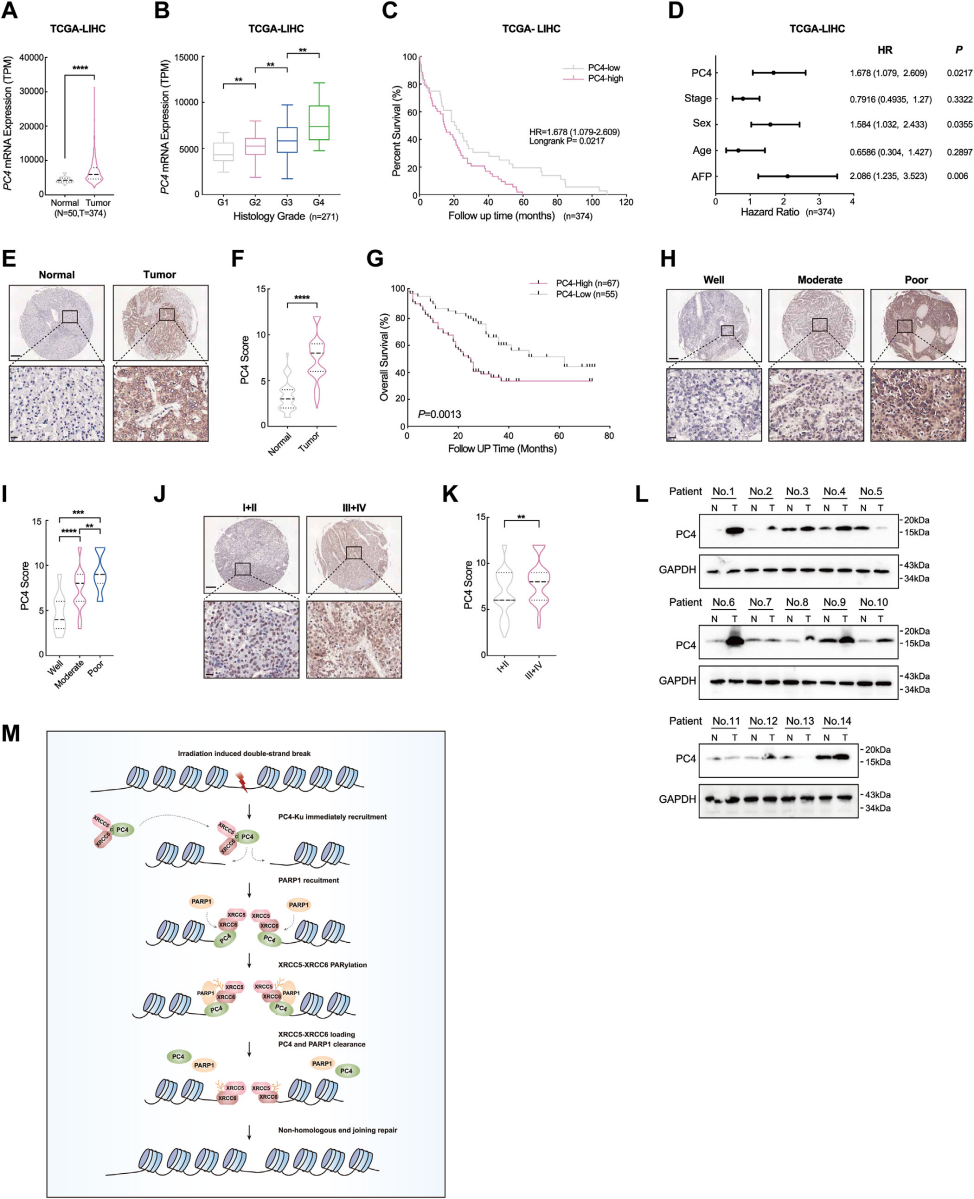
**PC4 levels might predict outcomes for patients with HCC.***A*, TCGA dataset analysis of PC4 mRNA expression in liver cancer patients (n = 374) as compared with healthy controls (n = 50). TPM, Transcripts Per Million. *B*, TCGA dataset analysis of PC4 mRNA expression in different histological stage of liver cancer patients (n = 271). *C*, TCGA dataset analysis of PC4 mRNA expression with overall survival in liver cancer patients (n = 374). Hazard ratio and *p*-values are indicated in the graph. *D*, multivariate Cox regression analysis of PC4 mRNA expression, sex, AFP levels, age, and tumor stage with overall survival in liver cancer patients (n = 374) in TCGA LICH dataset. *E*, representative images of PC4 immunostaining in human HCC (n = 122) and paired adjacent tissues (n = 111). Scale bar, *upper* = 200 μM, *lower* = 20 μM. *F*, PC4 IHC score in *E*. *G*, Kaplan–Meier survival curves of patients stratified by PC4 expression in *E*. *H*, representative images of PC4 immunostaining in well (n = 9), moderate (n = 90), and poor (n = 23) differentiated tissue samples. *Lower panels* (scale bar = 20 μM) show the enlarged sections of the *upper* ones (scale bar = 200 μM). *I*, PC4 IHC score of different histological stages in *H*. *J*, representative images of PC4 immunostaining different stages in liver cancer tissue microarray (I+II, n = 65; III+IV, n = 57). *upper panel* scale bar = 200 μM, *lower panel* scale bar = 20 μM. *K*, PC4 IHC score of different stages in *J*. *L*, Western blot analysis of PC4 protein level in 14 paired human HCC and adjacent tissue lysates. *M*, a proposed model illustrating that PC4 recruits PARP1 and facilitates XRCC5-XRCC6 complex PARylation, which initiates NHEJ repair. All quantifications are shown as mean ± SD. ∗∗*p* < 0.01, ∗∗∗*p* < 0.001, ∗∗∗∗*p* < 0.0001. HCC, hepatocellular carcinoma; NHEJ, nonhomologous end joining; PARP1, poly (ADP-ribose) polymerase 1; PC4, human positive cofactor 4.

Considering the role of PC4 in DNA damage repair, we investigated whether PC4 expression might have clinical significance in patients with HCC. To explore this possibility, the LICH-TCGA dataset, two human HCC tissue microarrays and 14 paired HCC-normal samples were used to measure PC4 mRNA and protein expression. The results revealed that PC4 was expressed at significantly higher levels in tumor tissues than in corresponding normal controls (Fig. 7, *A*, *E*, *F* and *L*, and Table S2). The higher the level of PC4 expression, the more advanced the clinical stage and the worse the prognosis of patients (Fig. 7, *B*–*D* and *G*–*K*). Based on the fact that the level of PC4 might predict the outcomes of patients with HCC and that PC4 can protect liver cancer cells from DNA damage, we propose that PC4 can be an effective biomarker and therapeutic target for radioresistant liver cancer.

## Discussion

PC4 has been reported to participate in DNA damage repair in distinct human cell types (22, 23, 25, 26, 27). Herein, our data revealed the essential role of PC4 in DSB repair *via* the NHEJ pathway in liver cancer cells. Previous studies have revealed that PC4 favors the NHEJ pathway because it upregulates the expression of nonhomologous end-joining factor 1 (25); however, compelling evidence has also suggested that PC4 promotes HR repair by interacting with replication protein A (27). In this study, we demonstrated that PC4 is recruited to DSB sites and associates with PARP1. This association serves as a key determinant of XRCC5 and XRCC6 PARylation and heterodimer loading onto DSB sites (Fig. 7*M*). Therefore, PC4 acts as a key docking site for the proper assembly of DDR-related proteins at DSBs, promoting efficient NHEJ repair.

Recruitment of XRCC5 and XRCC6 to DSBs has been identified as a crucial molecular event for determining whether NHEJ or HR is initiated as their recruitment promotes NHEJ initiation but suppresses DSB end resection–mediated HR repair (34). However, how XRCC5 and XRCC6 recruitment to DSBs is regulated remains unclear. Recently, several studies have documented that PARP1 promotes NHEJ repair through the recruitment and retention of PARylated XRCC5/XRCC6 at DSBs (18). However, it remains unclear whether additional molecules can modulate PARP1 activity and facilitate XRCC5/XRCC6 loading onto DSBs. The present study revealed that PC4 associates with PARP1 after IR-induced DNA damage and directs PARP1 function downstream of the Ku complex. Similar to PC4, PARP1 enhances DNA damage repair *via* HR (35). Therefore, we propose that the PC4-PARP1 complex provides a platform for coordinating the recruitment of key DNA repair proteins, such as Ku and possibly MRN. The initiation of a repair pathway may depend on the specificity of the substrates of the PC4–PARP1 complex, which may be determined by many factors, such as the source of DNA damage, cellular context, cell cycle progression, and its association with distinct partners, which should be investigated further. Considering the vital role of PC4 in DNA damage repair, small-molecule inhibitors targeting PC4 may block NHEJ repair and suppress cell resistance to IR, suggesting a possible intervention for tumor growth.

Radiotherapy is an essential therapeutic strategy for the treatment of HCC (2, 3). Nevertheless, resistance to radiotherapy still remains the primary cause of treatment failure and relapse in HCC. Therefore, identification of reliable biotargets that can be inhibited to sensitize HCC cells to radiotherapy is required to improve HCC treatment. Our findings showed that PC4 promotes NHEJ-prone DNA damage repair and renders HCC cells resistant to IR *in vitro and in vivo*. Additionally, patients with HCC having high PC4 expression are more likely to experience poor survival outcomes. Hence, we presume that PC4 is a promising gene that can be targeted to sensitize HCC cells to radiotherapy.

## Experimental procedures

### Human tissue specimens

Two human HCC tissue microarrays contained 122 HCC tissues, and paired 111 adjacent normal tissues were obtained from OUTDO Biotech. Fourteen frozen carcinoma tissues of HCC with paired adjacent normal tissues were obtained at affiliated hospital of Army Medical University. All patients provided informed consent, and all HCC tissues were diagnosed independently by at least two experienced pathologists, according to the Union for International Cancer Control classification system. The study was approved by the Ethics Committee of Third Military Medical University (Army Medical University) and abide by the Declaration of Helsinki principles.

### Plasmids

All the clone sequences and vectors information can be found in Table S3.

The lentiviral plasmid expressing shPC4 was generated by cloning shRNA targeting PC4 into the hU6-MCS-CBh-gcGFP-IRES-puromycin vector. The lentiviral plasmid expressing PC4-OV was generated by cloning PC4 corresponding coding sequences into a Ubi-MCS-3FLAG-SV40-EGFP-IRES-puromycin vector. The Flag-tagged PC4 deletion mutants (WT, Δ1, Δ2, Δ3, Δ4, Δ5, Δ6) were introduced by sequence synthesis, followed by cloning into pLentiPuro vectors. The His-GST-tagged-PC4 or His-GST-tagged-PC4-domains were generated by cloning the indicated sequence into GST-tagged vectors.

The plasmid expressing MYC-XRCC5 or HA-XRCC6 was constructed by cloning MYC-XRCC5-ORF or HA-XRCC6-ORF into CMV-MCS-EGFP-SV40-Neomycin vector. The plasmid expressing PC4-GFP or XRCC6-GFP was constructed by cloning PC4 or XRCC6 coding sequence into Ubi-MCS-EGFP-IRES-puromycin vector, respectively. All vectors constructed in this study were verified by Sanger sequencing before used.

### Cells

The sources of each cell line are listed in Table S3. All cells were cultured in Dulbecco’s modified Eagle’s medium (Gibco) supplemented with 10% fetal bovine serum (Biological Industries) and 1% penicillin/streptomycin (Beyotime) and maintained at 37 °C in a 5% CO2 atmosphere and routinely checked for *mycoplasma* contamination.

PC4 stably knockdown cell was generated by using shPC4 virus. The lentiviral vector was packaged with psPAX2 and pMD2. G and incubated with HEK293T for 48 h. Viral particles were collected, filtered, and transfected into expected cell lines to knockdown PC4. Subsequently, cells were selected by puromycin. PC4-knockdown efficiency was evaluated by Western blot.

PC4 stably overexpressing cell was generated by using PC4-OV virus. Cells were transduced with PC4-OV virus, and stable polyclonal populations of cells were selected by puromycin.

PC4-GFP labeled cell was generated by using the corresponding lentivirus. Fluorescent activated cell sorting was performed to isolate fluorescent populations.

Huh7-PARP1-knockout cell was generated by using CRISPR-Cas9. In brief, gene specific sgRNAs against PARP1 were cloned into the vector pX330-CRISPv2 cloning vector. Then, Huh7 cells were transfected with gRNA/Cas9 expression construct. After positive selection by using puromycin, cells were individually seeded in 96-well-plates. Monocolonies were grown until several weeks. PARP1-knockout efficiency was assessed by Sanger sequencing, and the disruption of PARP1 protein expression was evaluated by using Western blot. The sgRNA information can be found in Table S3.

### RNA interference

siRNAs targeting PC4, XRCC6 and PARP1 were respectively transfected into cells by using Lipofectamine RNAiMAX (Thermo Fisher Scientific) to transiently knockdown indicated gene. The knockdown efficiency was determined by Western blot. The siRNAs used are detailed in Table S3.

### Laser microirradiation

Huh7, HepG2, HeLa, and HEK293T cells were grown on glass-bottomed dishes and presensitized with 1 BrdU for 2 h. Subsequently, cultured cells were locally irradiated with a 365 nm UV microirradiation using a Zeiss Axiovert equipped with LSM 520 Meta. Subsequently, cells were fixed with 4% paraformaldehyde for 30 min at room temperature. After that, cells were incubated with 0.5% Triton X-100 for 5 min and blocking solution (Beyotime) for 30 min. Next, staining was performed with primary antibody overnight at 4 °C, followed by secondary antibody incubation and DAPI staining. The antibodies and dyes used are detailed in Table S3. Images were acquired using a Lecia SP-8 STED 3X microscope at room temperature.

For live imaging the recruitment of PC4 or XRCC6 to laser-induced DNA damage site, laser microirradiation was carried out on an OLYMPUS FV31S-SW confocal microscope equipped with an environmental chamber set to 37 °C. A 1 μm diameter band of damage was introduced across the width of the nucleus. Confocal images were recorded before and after laser irradiation every one second time intervals over a period of 30 min. The fluorescence intensity of the damaged region was then monitored over time after correcting for background and fluorescence loss.

### Immunofluorescence

Cells were plated into 20-mm glass bottom dishes the day before experiments. Then cells were treated with 4 Gy of X-ray irradiation (Rad source). After indicated time, cells were fixed with 4% paraformaldehyde, incubated with 0.5% Triton X-100, and blocked in blocking solution. Next, samples were incubated with anti-γH2AX overnight at 4 °C, followed by fluorochrome-conjugated secondary antibody incubation and DAPI staining. Images were acquired using a Lecia SP-8 STED 3X microscope at room temperature.

### Neutral comet assay

Comet assays were conducted according to the manufacturer’s instructions of the single-cell gel electrophoresis assay kit made by R&D systems (Catalog#4250-050-K). In brief, cells were treated with 2 Gy of X-ray irradiation (Rad source) and harvested either just after this treatment or after 5 min, 30 min, or 120 min of recovery. After electrophoresis, the slides were air-dried at room temperature. Individual cells were stained with SYBR Green I staining solution and viewed using a fluorescence microscope. The results were analyzed by CASP software, and at least six randomly slide per condition were selected for statistical analysis.

### NHEJ and HR reporter assays

NHEJ reporter plasmid EJ5-GFP (Addgene plasmid #44026) or HR reporter plasmid DR-GFP (Addgene plasmid #26475) was transfected into Huh7 cells respectively. Stable expression clones were selected using puromycin. Then cells were transfected with two different siRNAs to knockdown PC4. After that, cells were electroporated with pCBASce plasmid (Addgene plasmid #26477) at 270 V, 950 μF using a BioRad Genepulsar to induce double DNA breaks. After 48 h, cells were subjected to flow cytometry analysis to detect the percentage of GFP-positive cells, representing the efficiency of NHEJ or HR repair.

### Colony formation assay

Each group of Huh7 or HepG2 cells were seeded in 6-well plates at appropriate density. Cells were irradiated with different radiation dose of X-ray (0, 2, 4, and 6 Gy) with three replicates. Colonies were formed after 2 to 3 weeks culturing. Cells were fixed in 4% paraformaldehyde for 5 min at room temperature, subjected to stain with 0.5% crystal violet for 15 min, and washed with PBS. The images were automatically captured and colonies consisting over 50 cells were counted. Plate clone formation efficiency = (number of colonies/number of cells inoculated) × 100%. Survival fractions (SFs) were calculated by normalizing the data to the plating efficiency of appropriate control groups. The survival curve was derived from a multitarget single-hit model: SF = 1-(1-exp(-D/D0))n. D0 was defined as the dose that gave an average of one hit per target. The radiation sensitivity enhancement ratio was measured according to the multitarget single-hit model.

### Xenograft model and radiotherapy

For *in vivo* xenograft model, 6-weeks-male BALB/c nude mice (Gempharmatech) were fed in a specific pathogen-free room and inoculated subcutaneously with each group of Huh7 cells (5 × 106 in 200 μl PBS) at one dorsal site. When the tumor size reached a volume of 100 mm^3^, nude mice harboring Huh7 (shPC4, PC4-OV or controls) xenografts were treated with X-rays (2 Gy/day for 5 consecutive days). Only tumor locations were exposed to ionizing radiation, and the other mouse body was shielded with a lead plate. Tumor size was monitored by calipers every 4 days, and tumor volumes were calculated as length × (width)^2^/2. At the endpoint, the mice were sacrificed, and then xenografts from animals were isolated and weighted. Subsequently, tumors and organs were dissected to be fixated by 4% paraformaldehyde for further analysis. All the animal experiments were carried out with the approval of the Ethics Committee of Third Military Medical University (Army Medical University).

### Immunoprecipitation

Cells were collected and lysed by using IP lysis buffer (50 mM Tris-HCl pH 7.5, 1 mM EDTA, 150 mM NaCl, 1 mM EGTA, 5 mM MgCl2, 10%glycerol and 0.2%NP-40), supplemented with protease inhibitor (Thermo Fisher Scientific), phosphatase inhibitors cocktail 2 (Sigma-Aldrich, P5726), and 1 mM 1,4-dithiothreitol (Sigma-Aldrich). Then cell lysate was centrifuged 20000*g* for 15 min at 4 °C, and the supernatant was incubated with primary antibody for 4 h at 4 °C. After that, the antibody-bound protein was subjected to incubate with Protein A/G Beads (ThermoFisher, 88802) for 2 h at 4 °C. After six washes with wash buffer (0.5 M Tris-HCl pH 7.4, 1.5 M NaCl), immunoprecipitated complexes were denatured with loading buffer (Fdbio science) for 10 min at 95 °C. The samples were then stored at −20 °C or ready for SDS-PAGE (sodium dodecyl sulfate-polyacrylamide gel electrophoresis).

### Proximity ligation assay

Huh7 cells were fixed with 4% paraformaldehyde and permeabilized with 0.5% Triton X-100, followed by incubation with primary antibodies for PC4 and XRCC5 or XRCC6. PLA was performed using the Duolink PLA kit (DUO92101, Sigma-Aldrich) according to the manufacturer’s instructions. The specificity of the primary antibodies was confirmed by siRNAs-mediated knockdown followed by Western Blot analysis.

### Sucrose gradient fraction

Huh7 cells were harvested and lysed by NETN buffer. Subsequently, the cell lysate was centrifuged at 14,000 rpm for 10 min at 4 °C (Optima L-100 XP Ultracentrifuge). The supernatant was loaded onto the sucrose cushion and centrifuge at 36,000 rpm for 3 h at 4 °C with SW41 Tt rotor. After that, each fraction was collected by fractionation system, and proteins were isolated for further analysis.

### Western blot analysis

Protein extracts were separated by SDS-PAGE and electrophoretically transferred to 0.45 μm PVDF membrane (EMD Millipore). The membrane was incubated for 1 h in 5% milk/PBST at room temperature, followed by cut into strips corresponding to the target’s molecular mass. Then, the strips were incubated with indicated primary antibodies at 4 °C overnight. After three washes with three times of PBST, the membrane strips were incubated with appropriate secondary antibodies conjugated to horseradish peroxidase at 1:1000 dilution in 5% milk/PBST. Membranes were visualized using the Immobilon Western Chemiluminescent HRP Substrate (EMD Millipore) in the ImageQuant LAS 4000 platform or Bio-Rad ChemiDoc.

### Mass spectrometry

Cells lysates were incubated with PC4 antibody, and immunoprecipitated protein were reduced with 50 mM dithiothreitol (Sigma-Aldrich) at 56 °C for half 1 h. After that, the proteins were alkylated with 11 mM iodoacetamide (Acros) at room temperature in the darkness for 15 min. Subsequently, the precipitation was digested by incubation with 0.01 μg/μl trypsin (Promega) at 37 °C overnight. Next, the peptides were desalted by C18 Ziptips and analyzed by the liquid chromatography–tandem mass spectrometry (LC-MS/MS) on Thermo ScientificTMEASY-nLCTM 1000. The LC-MS/MS data were processed using Proteome Discoverer (Thermo Fisher Scientific) and searched against the Swiss-Prot *Homo sapiens* protein sequence database.

### GST pulldown assay

Recombinant GST-tagged PC4 proteins were purified from bacteria and recombinant XRCC5/XRCC6 heterodimer protein were bought from Sino Biological (Catalog#CT018-H07B). For GST pulldown assay, recombinant GST-tagged PC4 protein and recombinant XRCC5/XRCC6 heterodimer protein were co-incubated for 4 h at 4 °C followed by glutathione-agarose beads (GE Healthcare) incubation at 4 °C for an additional 2 h on a rotating wheel. Beads were then washed with NETN buffer three times, and bound proteins were analyzed by Western blotting.

### Statistical analysis

Statistical analysis was carried out using SPSS 22.0 and GraphPad Prism software, and all data are presented as means ± SD. Comparisons between two groups were performed using unpaired Student’s *t* test. Comparisons among three or more groups were performed using a one-way analysis of variance (ANOVA). The survival data were performed using the Kaplan–Meier method. Correlation between PC4 expression and clinical parameters was determined using the Pearson’s χ2 method. *p* < 0.05 indicated a statistically significant difference.

### Reagents

Information of all the reagents used in this study are described in Table S3.

## Data availability

The data that support the findings of this study are available from the corresponding author upon request. Data are available *via* ProteomeXchange with identifier PXD042218.

## Supporting information

This article contains supporting information.

## Conflict of interest

All authors declare no conflict of interest with the contents of this article.

## Supporting information

Supporting Table S3

Supporting Figure S1 **A widespread effect of PC4 on DNA damage response.** Related to Figure 2 (*A*) Western blot analysis of the expression of γ-H2AX in HepG2 cells with PC4 knockdown upon irradiation. *B*, Western blot analysis of the expression of ATR, p-ATR, ATM, p-ATM, CHK1, p-CHK1, CHK2, p-CHK2, DNA-PKcs and p-DNA-PKcs and in Huh7 and HepG2 cells with PC4 knockdown and irradiation at 4 Gy.

Supporting Figure S2 **PC4 physically associates with Ku complex through C-terminal domain.** Related to Figure 3 (*A*) IP analysis of the association of PC4 with XRCC5 and XRCC6 in HeLa and 293T cells. *B*, reciprocal co-IP analysis of the association of XRCC5 with PC4 and XRCC6 in HepG2 cells. C, selected confocal images of Huh7 transfected with Flag-tagged PC4 truncated mutants. Scale bar = 4 μm.

Supporting Figure S3 **PC4 is required for XRCC6 PARylation at DSB sites.** Related to Figure 4 (*A*) IP analysis of the association of Flag-tagged PC4 with HA-tagged XRCC6 in Huh7 cells after 3 Gy irradiation. *B*, IP analysis of the association of Flag-tagged PC4 with MYC-tagged XRCC5 in Huh7 cells after 3 Gy irradiation. *C*, reciprocal co-IP analysis of the association of endogenous XRCC6 with PC4 and XRCC5 in Huh7 cells after 3 Gy irradiation. *D*, reciprocal co-IP analysis of the association of HA-tagged XRCC6 and Flag-tagged PC4 in Huh7 cells after 3 Gy irradiation. *E*, reciprocal co-IP analysis of the association of MYC-tagged XRCC5 and Flag-tagged PC4 in Huh7 cells after 3 Gy irradiation.
