# Supplementary figures and images for: PC4-mediated Ku complex PARylation facilitates NHEJ-dependent DNA damage repair
Source: J Biol Chem. 2023 Jul 10;299(8):105032. doi: 10.1016/j.jbc.2023.105032 (PMC10406618; doi:10.1016/j.jbc.2023.105032)

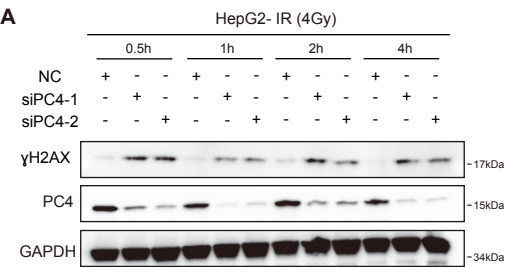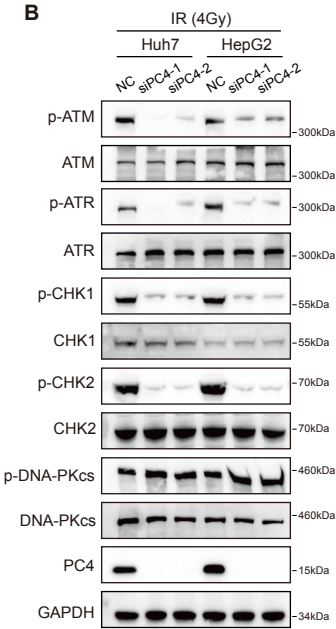

Supplement: Supporting Figure S1 — A widespread effect of PC4 on DNA damage response. Related to Figure 2 (A) Western blot analysis of the expression of γ-H2AX in HepG2 cells with PC4 knockdown upon irradiation. B, Western blot analysis of the expression of ATR, p-ATR, ATM, p-ATM, CHK1, p-CHK1, CHK2, p-CHK2, DNA-PKcs and p-DNA-PKcs and in Huh7 and HepG2 cells with PC4 knockdown and irradiation at 4 Gy. [file mmc4.pdf]

**A**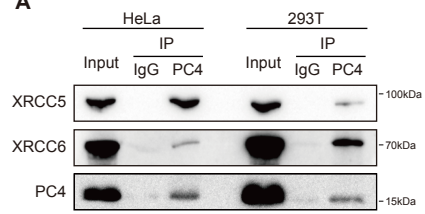**B**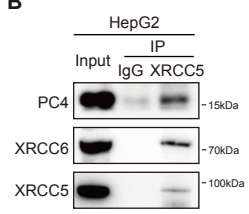**C**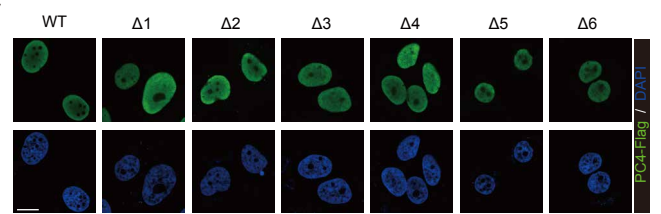

Supplement: Supporting Figure S2 — PC4 physically associates with Ku complex through C-terminal domain. Related to Figure 3 (A) IP analysis of the association of PC4 with XRCC5 and XRCC6 in HeLa and 293T cells. B, reciprocal co-IP analysis of the association of XRCC5 with PC4 and XRCC6 in HepG2 cells. C, selected confocal images of Huh7 transfected with Flag-tagged PC4 truncated mutants. Scale bar = 4 μm. [file mmc5.pdf]

**A**

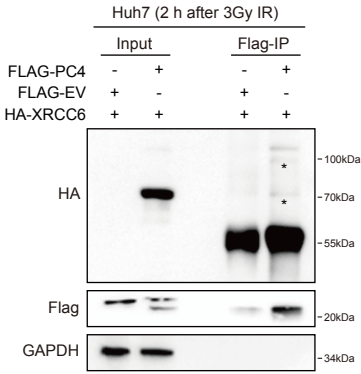

**B**

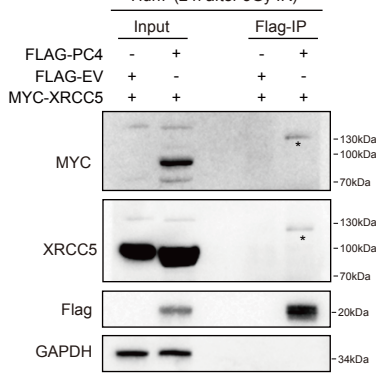

**C**

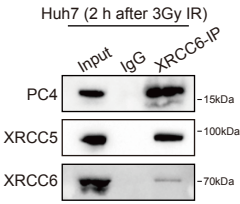

**D**

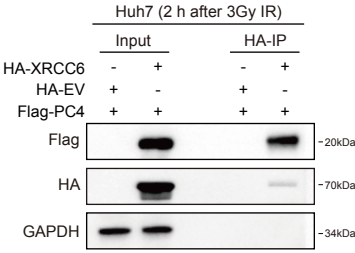

**E**

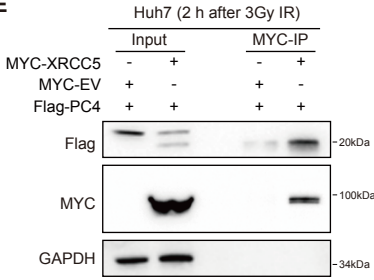

Supplement: Supporting Figure S3 — PC4 is required for XRCC6 PARylation at DSB sites. Related to Figure 4 (A) IP analysis of the association of Flag-tagged PC4 with HA-tagged XRCC6 in Huh7 cells after 3 Gy irradiation. B, IP analysis of the association of Flag-tagged PC4 with MYC-tagged XRCC5 in Huh7 cells after 3 Gy irradiation. C, reciprocal co-IP analysis of the association of endogenous XRCC6 with PC4 and XRCC5 in Huh7 cells after 3 Gy irradiation. D, reciprocal co-IP analysis of the association of HA-tagged XRCC6 and Flag-tagged PC4 in Huh7 cells after 3 Gy irradiation. E, reciprocal co-IP analysis of the association of MYC-tagged XRCC5 and Flag-tagged PC4 in Huh7 cells after 3 Gy irradiation. [file mmc6.pdf]
